# Is There an Association Between Cortisol and Hypertension in Overweight or Obese Children?

**DOI:** 10.4274/jcrpe.4802

**Published:** 2017-12-15

**Authors:** Aleid JG Wirix, Martijn JJ Finken, Ines A von Rosenstiel-Jadoul, Annemieke C Heijboer, Jeroen Nauta, Jaap W Groothoff, Mai JM Chinapaw, Joana E Kist-van Holthe

**Affiliations:** 1 VU University Medical Center, EMGO Institute for Health and Care Research, Department of Public and Occupational Health, Amsterdam, The Netherlands; 2 VU University Medical Center, Department of Pediatric Endocrinology, Amsterdam, The Netherlands; 3 MC Slotervaart Hospital, Clinic of Pediatrics, Amsterdam, The Netherlands; 4 VU University Medical Center, Department of Clinical Chemistry, Endocrine Laboratory, Amsterdam, The Netherlands; 5 Erasmus MC Sophia Children’s Hospital, Clinic of Pediatric Nephrology, Rotterdam, The Netherlands; 6 Emma Children’s Hospital/Academic Medical Center, Department of Pediatric Nephrology, Amsterdam, The Netherlands

**Keywords:** Hypertension, obesity, children, cortisol, pathophysiology

## Abstract

**Objective:**

The precise mechanisms behind the development of hypertension in overweight or obese children are not yet completely understood. Alterations in hypothalamic-pituitary-adrenal axis activity may play a role. We aimed to investigate the association between cortisol parameters and hypertension in overweight or obese children.

**Methods:**

Random urine (n=180) and early-morning saliva samples (n=126) for assessment of cortisol and cortisone were collected from 1) hypertensive overweight children (n=50), 2) normotensive overweight children (n=145), and 3) normotensive non-overweight children (n=75).

**Results:**

The age of participants was 10.4±3.3 years and 53% were boys. The urinary cortisol-to-cortisone ratio [β 1.11, 95% confidence interval (CI) 1.05-1.19] as well as urinary cortisol/creatinine (β 1.38, 95% CI 1.09-1.54), and cortisone/creatinine ratios (β 1.26, 95% CI 1.17-1.36) were significantly higher in overweight or obese than in non-overweight children. After adjusting for body mass index-standard deviation score and urinary cortisone/creatinine ratio, but not cortisol/creatinine ratio, was significantly associated with presence of hypertension (β 1.12, 95% CI 1.02-1.23). Salivary cortisol and cortisone levels were significantly lower in overweight or obese than in non-overweight children (β -4.67, 95% CI -8.19- -1.15, and β 0.89, 95% CI 0.80-0.97 respectively). There were no significant differences in cortisol parameters between hypertensive and normotensive overweight or obese children.

**Conclusion:**

This study provided further evidence for an increased cortisol production rate with decreased renal 11β-hydroxysteroid dehydrogenase 2 activity and flattening of early-morning peak cortisol and cortisone in overweight or obese children. However, there were no significant differences in cortisol parameters between hypertensive and normotensive overweight and obese children.

**What is already known on this topic?**

It is known that obesity can lead to hypertension. However, the precise mechanisms behind the development of hypertension in overweight or obese children are not yet completely understood.

**What this study adds?**

There is evidence for an increased cortisol production rate with decreased renal 11β-hydroxysteroid dehydrogenase 2 activity and flattening of early-morning peak cortisol and cortisone in overweight or obese children. There is no evidence for a role for cortisol in hypertension-induced obesity.

## INTRODUCTION

As a result of the growing overweight and obesity epidemic, hypertension is increasingly common, even in childhood; 4–14% of overweight children and 11–33% of obese children are diagnosed with hypertension (1,2,3). Since both overweight and hypertension have a tendency to track from childhood into adulthood, these findings are of great concern (4,5).

Establishing the cause of hypertension in obese children is of utmost importance for the development of therapeutic strategies. However, the pathophysiology of hypertension in obesity is complex and not fully understood (6). Several studies suggest that alterations in the production and/or metabolism of glucocorticoids could play a role in the pathophysiology of the metabolic syndrome (7,8), given its phenotypic similarities with Cushing’s syndrome (9,10). Glucocorticoids stimulate hepatic glucose production, lipolysis, vascular reactivity, and sodium reabsorption (11).

The tissue effects of glucocorticoids are for an important part regulated by 11β-hydroxysteroid dehydrogenase (11β-HSD) isozymes, which interconvert cortisol with its inert metabolite cortisone. There are two isozymes. Type 1 generates cortisol from cortisone and is expressed mainly in liver and adipose tissue, and type 2 catalyses the reverse reaction primarily in the kidney. Pharmacological inhibition of renal 11β-HSD2 activity, e.g. by heavy use of liquorice, leads to hypertension by exposure of renal mineralocorticoid receptors to excess cortisol concentrations. The role of 11β-HSD type 1 in blood pressure regulation and hypertension is less well understood (7,8,12).

There are few studies in children on 11β-HSD activity in obesity-induced hypertension (12,13,14). One case study of four 10-to-15-year-old hypertensive obese boys found excess urinary adrenal androgen and cortisol metabolites (13). Another study in children aged 14 to 15 years, that made comparisons between obese children with (n=15) and without hypertension (n=11), and normotensive normal-weight children (n=15), found that the cortisol-to-cortisone ratio was higher in the hypertensive obese group than in the other two groups. Systolic blood pressure was positively associated with urinary tetrahydrocortisol (THF) +5α-THF/THE (allo-THF/tetrahydrocortisone) ratio, indicative of a cortisol/cortisone shuttle that favours cortisol (12). Another study found that serum adrenocorticotropic hormone (ACTH) and cortisol levels were positively associated with blood pressure in obese children aged 4 to 18 years, yet there was no control group. These results suggest that the hypothalamic-pituitary-adrenal (HPA) axis is involved in the development of obesity-induced hypertension in children (14).

With the present study, we aimed to investigate if there is an association between cortisol parameters, including cortisol and cortisone in early-morning saliva and in random urine collections, and hypertension in overweight and obese children. To disentangle the contributions of hypertension and being overweight, we included two control groups, namely normotensive overweight and obese children and normotensive non-overweight children.

## METHODS

### Population and Design

Non-fasting urine and early-morning fasting saliva samples were collected from a convenience sample of Dutch children aged 5 to 17 years, in the period between September 2013 and June 2015, consisting of: 1) n=50 overweight and obese children with hypertension, 2) n=145 overweight and obese children without hypertension, and 3) n=75 non-overweight children without hypertension. In total, 36 children provided both urine and saliva samples, 141 children provided only urine samples, and 90 children only saliva samples.

Overweight and obese children with and without hypertension were recruited at a pediatric outpatient obesity clinic and through their participation in our study on the prevalence of hypertension in overweight and obese children (15). The control group of healthy non-overweight children was recruited at a general pediatric outpatient clinic, which they visited for various reasons, and at two schools. Children with conditions that might affect blood pressure, for example a history of urinary tract infections, were not eligible for inclusion.

The study protocol has been approved by the VU University Medical Center Ethics Committee (approval nuber: A2015,121). Informed consent was obtained from at least one of their parents and from all children above the age of 12 years.

### Anthropometry and Blood Pressure Measurements

Height was measured to the nearest 0.1 centimetre using a stadiometer. Body weight was measured to the nearest 0.1 kilogram using a digital balance scale with children barefooted and wearing light clothing. Body mass index (BMI) was calculated as weight in kilograms divided by the square of body height in meters and categorized according to the International Obesity Task Force (16).

Blood pressure was measured three consecutive times at the right arm after 5 minutes of rest in sitting position using an electronic oscillometric blood pressure device. An appropriate-sized cuff was used according to the guidelines of the National High Blood Pressure Education Program (NHBPEP) Working Group on Children and Adolescents (17). Based on the lowest of three consecutive blood pressure measurements, hypertension was defined as ≥95th percentile for age, gender, and height (17).

### Urine and Saliva Sample Analyses

Urine samples were collected on site. Early morning saliva samples were obtained using a Salivette® (Sarstedt AG & Co. Nümbrecht, Germany) swap which was provided during the visit together with a return envelope. Participants were requested to obtain saliva immediately after awakening, between 06.00 and 09.00 a.m. and prior to having breakfast and to return the sample by postal mailing.

Urine and saliva were stored at -80 °C. Both samples were analysed for cortisol and cortisone. 0.1 mL of urine or 0.1 mL of saliva was used to assess cortisol and cortisone concentrations, using an isotope dilution liquid chromatography-tandem mass spectrometry (LC-MS/MS) method. Internal standards (13C3 labeled cortisol and cortisone) were added to the samples. Samples were extracted using supported liquid extraction (Isolute, Biotage, Uppsala, Sweden) and analysed by LC-MS/MS [Quattro Premier XE tandem mass spectrometer (Waters Corp., Milford, Massachusetts, USA)]. Lower limit of quantitation was 1.0 nmol/L for cortisol and 0.5 nmol/L for cortisone. The intra-coefficients of variation (CV%) for cortisol were 7 and 4% at a level of 3 and >5 nmol/L, respectively, and for cortisone <5% at all levels >2.8 nmol/L. The inter-CV% was <11% for both cortisol and cortisone.

### Outcome Measures

Cortisol/creatinine ratio in spot urine is a measure of cortisol production (18). The urinary cortisol-to-cortisone ratio reflects renal 11β-HSD2 activity (12,19). Cortisol and cortisone in early-morning saliva are indicators of the morning peak in HPA axis activity (20). The salivary cortisol-to-cortisone ratio is only a rough estimate of the systemic interconversion between 11β-HSDs.

### Statistical Analysis

BMI and height standard deviation scores (SDSs) were calculated using the LMS method (16), based on reference values from the World Health Organization (21) and Centers for Disease Control, respectively (22). Blood pressure SDSs were calculated using the equations provided by the NHBPEP Working Group (17). Differences in characteristics between hypertensive overweight and obese children, normotensive overweight and obese children, and normotensive non-overweight children were tested with ANOVA and post-hoc t-tests. Linear regression analysis was used to test associations with cortisol parameters between children with and without hypertension, adjusted for BMI-SDS, and between overweight and non-overweight children, adjusted for blood pressure SDS. Results are expressed as beta coefficients with 95% confidence intervals (95% CI). A p-value <0.05 was considered statistically significant. The statistical analyses were performed with SPSS software version 22.0 (SPSS Inc., Chicago, Illinois).

### RESULTS

A total of 270 children and adolescents were included in the study. Urine samples were collected from 180 children (38 hypertensive overweight and obese children, 86 normotensive overweight and obese children, and 56 normotensive non-overweight children), and saliva samples from 126 children (17 hypertensive overweight and obese children, 64 normotensive overweight and obese children, and 45 normotensive non-overweight children). Demographic, anthropometric, and blood pressure data are presented in Table 1.

Salivary and urinary cortisol and cortisone, and cortisol-to-cortisone ratios are displayed in Table 2.

Salivary cortisol and cortisone levels, but not the salivary cortisol-to-cortisone ratio, were significantly lower in overweight or obese children than in non-overweight children (β 0.89, 95% CI 0.80-0.97, and β -4.67, 95% CI -8.19- -1.15, respectively). There were no significant differences in these parameters between hypertensive and normotensive children. Salivary cortisol levels were not significantly associated with systolic (β 0.61, 95% CI 0.31-1.21) or diastolic (β 1.20, 95% CI 0.74-1.97) blood pressure SDS.

Urinary cortisol/creatinine (β 1.38, 95% CI 1.09-1.54) and cortisone/creatinine ratios (β 1.26, 95% CI 1.17-1.36), and the cortisol-to-cortisone ratio (β 1.11, 95% CI 1.05-1.19) were significantly higher in overweight or obese than in non-overweight children. Urinary cortisol/creatinine (β 1.20, 95% CI 1.06-1.36) and cortisone/creatinine ratios (β 1.19, 95% CI 1.08-1.30), but not the urinary cortisol-to-cortisone ratio (β 1.02, 95% CI 0.95-1.09), were higher in hypertensive children than in normotensive children. After adjustment for BMI-SDS, the association between urinary cortisol/creatinine ratio and hypertension was no longer significant (β 1.11, 95% CI 0.97-1.25), but the association between cortisone/creatinine ratio and hypertension remained significant (β 1.12, 95% CI 1.02-1.23). After adjustment for BMI-SDS, urinary cortisol/creatinine ratio was not significantly associated with systolic (β 1.42, 95% CI 0.91-2.21) or diastolic (β 1.30, 95% CI 0.94-1.81) blood pressure SDS.

## DISCUSSION

This study provided evidence for an increased renal excretion of free cortisol and cortisone, with higher excretion of cortisol relative to cortisone, in overweight or obese children. It also showed that early morning salivary levels of cortisol and cortisone were lower in overweight or obese children. However, there were no differences in cortisol parameters between hypertensive and normotensive overweight or obese children.

The findings from this study confirm previous observations (20,23) that childhood obesity is associated with an increased cortisol production rate, decreased renal 11β-HSD2 activity, and flattening of early-morning peak cortisol and cortisone. We found no significant differences in cortisol parameters between hypertensive and normotensive overweight or obese children. This is in contrast to previous studies in obese children which showed a positive association of systolic blood pressure, as part of metabolic syndrome, with increased serum levels of cortisol and ACTH (14,24), and in free cortisol in 24-hr urine (25). An explanation for the lack of an association with hypertension in our study is that the groups of hypertensive and normotensive overweight or obese children might have been too similar regarding presence of features of metabolic syndrome, as an index of glucose tolerance was not tested, to be able to detect differences in cortisol parameters.

In the total sample of overweight and non-overweight children, we found significant associations between ratios of urinary cortisol or cortisone to creatinine and the presence of hypertension. However, after adjustment for BMI-SDS, both associations became weaker, and only the association with urinary cortisone/creatinine ratio remained significant.

Future studies should elucidate whether hypertensive and normotensive overweight or obese children differ in the metabolism of cortisol. Cortisol is metabolized reversibly by 11β-HSDs and irreversibly by A-ring reductases and CYP3A4. In adults, impaired metabolic clearance of cortisol has been implicated to play a role in metabolic disease susceptibility (26).

A major strength of our study is inclusion of three study groups consisting of hypertensive overweight and obese children, normotensive overweight and obese children, and normotensive non-overweight children. This approach enabled us to study the relative contributions in overweight or obesity and hypertension. Another strength is the method we used to measure cortisol and cortisone concentrations. LC-MS/MS is known to be a very accurate, specific and sensitive method to measure steroid hormones (27,28).

### Study Limitations

Our study has several limitations. First, hypertension was based upon blood pressure measurements obtained on only one occasion, although three times consecutively. No 24-hour ambulatory blood pressure monitoring was performed to confirm the diagnosis of hypertension. Second, for practical reasons, only random daytime urine samples (with unspecified sampling times) were collected instead of 24-hr urine, although cortisol/creatinine ratio in spot urine has proven to be a reliable tool for the assessment of cortisol production (18). Third, only early-morning saliva samples were collected, so that association with diurnal rhythmicity in HPA axis activity could not be tested. Cortisol in scalp hair - as an index of long-term glucocorticoid exposure - was not tested in our sample, although recent studies have shown strong associations with indices of obesity (29). Yet another limitation is the cross-sectional study design of our study. A longitudinal study is necessary to gain insight into temporal relations. Ideally, the role of cortisol in the development of obesity-induced hypertension should be studied in a prospective cohort study with participants being sampled prior to developing overweight.

## CONCLUSION

We found that overweight and obese children had an increased cortisol production rate. Furthermore, overweight and obesity were associated with a higher urinary cortisol-to-cortisone ratio, reflecting decreased renal 11β-HSD2 activity, as well as with lower levels of early-morning cortisol and cortisone. However, there were no significant differences in cortisol parameters between hypertensive and normotensive overweight and obese children. More research is needed to elucidate whether cortisol metabolism is involved in the pathogenesis of obesity-induced hypertension in children.

## Figures and Tables

**Table 1:**
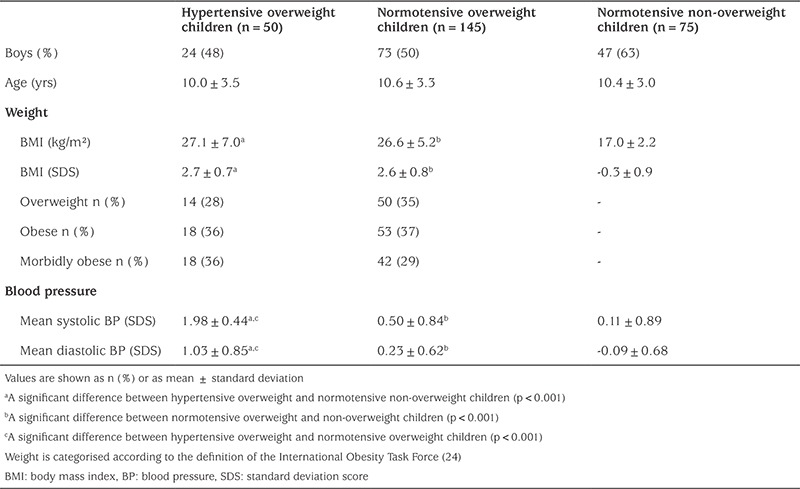
Characteristics of the study sample divided into hypertensive overweight, normotensiveoverweight, and normotensive non-overweight categories

**Table 2:**
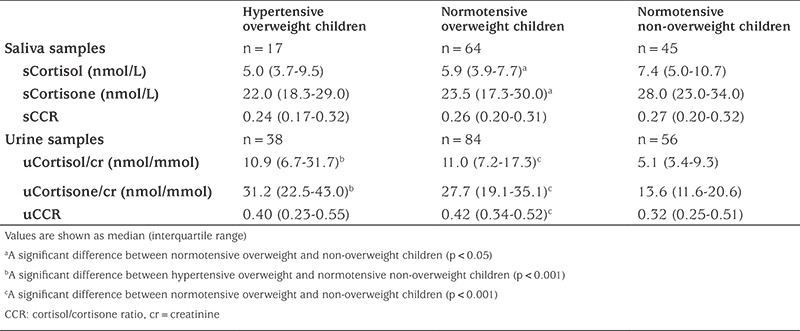
Salivary and urinary levels of cortisol and cortisone and cortisol/cortisone ratios in the subjects classified as hypertensive overweight, normotensive overweight, and normotensive non-overweight
